# 

**Published:** 1913-04-05

**Authors:** 


King's College Hospital.

Mr. Alfred Chune Fletcher, of the Charterhouse, has died at the age of fifty-eight. Educated at St. Bartholomew's Hospital in 1877 and subsequently senior house surgeon, Mr. Fletcher became well-known as a consulting physician in London. He qualified M.R.C.S. in 1881, and was resident medical officer at Sutton's Hospital in Charterhouse. He leaves a widow and three daughters—Miss Diana Chune Fletcher, Mrs. Arthur Michael Samuel, and Mrs. Arthur Howard.

### An Original Summary of Hormone Therapy.

For some time past our medical contemporary the *Prescriber* has been issuing special numbers, the original feature of which has been their exclusive devotion to some new development of therapeutics, or to some question on which medical opinion is still in a state of flux. This month a special double number is being issued, price one shilling (post free), dealing with Hormone therapy, a subject the literature of which has not yet been summarised adequately in the medical press. The history and possibilities of Hormone therapy are dealt with in a special article, while the general aspects of the subject are thrown into high relief by the contributions of a group of specialists in the subject. A bibliographical summary, a list of Hormone remedies, and a number of reviews complete an issue which is likely to remain for some time a convenient encyclopædia of this subject. It can be obtained from the Manager, 137 George Street, Edinburgh.

### LARGE REVERSION TO LONDON HOSPITALS.

Mr. Eaton John Caldbeck, wine merchant, of Messrs. Macgregor, Caldbeck and Co., has left various legacies, and, subject to his wife's life interest, the ultimate residue of his property equally between King's College Hospital, the Royal Free Hospital, the Free Cancer Hospital, Fulham Road, the Royal London Ophthalmic Hospital, City Road, the Cripples' Home and College, Alton, Hants, and the National Industrial Home for Crippled Boys, Wright's Lane, Kensington. The amount of this reversion will vary from about £10,000 to £30,000, according to the extent to which Mrs. Caldbeck exercises her power of appointment.

### Appointments.

Mr. Frederic Hibbert Westmacott, F.R.C.S. (Eng.), L.R.C.P. (Lond.), B.Sc. (Vict.), has been appointed honorary assistant aural surgeon to the Manchester Royal Infirmary.

At a special meeting of the board held on Wednesday, last week, Sir Edward Wood, J.P., was elected chairman of the board of governors of Leicester Royal Infirmary for the twelfth successive year.

At a meeting of the special elective committee of the Derbyshire Royal Infirmary, for the purpose of electing an additional honorary anæsthetist, Arthur Douglas Hunt, M.D., Ch.B. Liverpool, was appointed.

Mr. Owen Herbert Peters, Mr. Edward James Boome, and Miss Elizabeth Maud McVail have been appointed temporary medical assistants in the Public Health Department of the London County Council at a salary of £400 a year.

Dr. Adam White, M.B., B.S.Edin., lately assistant medical officer of the Sheffield City Hospital, has been appointed assistant tuberculosis medical officer for Herefordshire. He has held appointments at Edinburgh Royal Infirmary, and has had experience of the administrative treatment of tuberculosis at two Sheffield institutions.

### GLASGOW MATERNITY AND WOMEN'S HOSPITAL.

At a meeting of directors, held on March 10, Dr. James E. Paterson and Dr. John P. McVey were appointed indoor house surgeons at the hospital for six months from April 5 next; and Dr. Jane M. Davidson was appointed house surgeon at the West End Branch for three months from same date.

### Personalia.

St. Mary's Infirmary, Highgate Hill, recently lost one of the most valued members of its staff by the death of Sister Stanley Crawford. She came to the infirmary ten years ago from Bombay, where she spent her childhood, though she was actually a native of Highgate. She received her training at the infirmary, and, after passing some time as staff nurse, became ward sister. "She was," writes one who knew her well, "of a quiet and retiring disposition, and, though devoted to her work and a firm disciplinarian, she endeared herself to her subordinates and her patients alike, to the latter especially by her untiring efforts to ameliorate their lot. She died of pulmonary tuberculosis after a lingering illness of four months, borne with the greatest patience and fortitude." She was buried at Finchley, and her funeral was attended by a large number of the staff, including several past members of the medical staff, by whom she was held in great respect.

